# The effect of age on resilience of health-related quality of life among polytrauma patients: a cross-sectional multicenter study

**DOI:** 10.1007/s00068-022-02135-2

**Published:** 2022-11-22

**Authors:** Rob de Vries, Inge Reininga, Max de Graaf, Hester Banierink, Eelke Bosma, Arvid Munzebrock, Erik Heineman, Mostafa El Moumni, Klaus Wendt

**Affiliations:** 1 Department of Trauma Surgery, University Medical Center Groningen (UMCG), Groningen, The Netherlands; 2 Emergency Care Network Northern Netherlands, AZNN, Netherlands Trauma Registry, Northern Groningen, The Netherlands; 3 Department of Trauma Surgery, Martini Hospital (MZH), Groningen, The Netherlands; 4 Department of Trauma Surgery, Medical Centre Leeuwarden (MCL), Leeuwarden, The Netherlands; 5 Department of Surgery, University Medical Center Groningen (UMCG), Groningen, The Netherlands

**Keywords:** Polytrauma, Elderly, Health-related quality of life, Patient-reported outcome measures, Resilience

## Abstract

**Purpose:**

The aim of this study was to determine the impact of age on patient-reported health-related quality of life (HRQoL) and the capacity to show resilience—i.e., the ability to adapt to stressful adverse events—after sustaining a polytrauma.

**Methods:**

A cross-sectional multicenter cohort was conducted between 2013 and 2016 that included surviving polytrauma patients (ISS ≥ 16). HRQoL was obtained by the Short Musculoskeletal Function assessment and EuroQol (SMFA and EQ-5D-5L). The effect of age on HRQoL was tested with linear regression analysis. Next, the individual scores were compared with age- and sex-matched normative data to determine whether they showed resilience. Multivariate binary logistic regression was used to assess the effect of age on reaching the normative threshold of the surveys, correcting for several confounders.

**Results:**

A total of 363 patients responded (57%). Overall, patients had a mean EQ-5D-5L score of 0.73. With higher age, scores on the SMFA subscales “upper extremity dysfunction,” “lower extremity dysfunction” and “daily activities” significantly dropped. Only 42% of patients were classified as being resilient, based on the EQ-5D-5L score. Patients aged 60–69 showed the highest resilience (56%), and those aged 80 + showed the lowest resilience (0%).

**Conclusion:**

Sustaining a polytrauma leads to a serious decline in HRQoL. Aging is associated with a decline in the physical components of HRQoL. No clear relationship with age was seen on the non-physical components of quality of life. Octogenarians, and to a lesser extent septuagenarians and tricenarians, showed to be very vulnerable groups, with low rates of resilience after surviving a polytrauma.

## Introduction

Being polytraumatized, generally defined as having an injury severity score (ISS) of 16 and above, has a large impact on one’s life. Sustaining a polytrauma has globally been recognized as one of the leading causes of mortality, morbidity, occupational disability and loss of health-related quality of life (HRQoL) within the young population [1–5]. The effect of polytrauma on quality of life in surviving elderly remains unclear though.

Older polytrauma patients are a growing population representing almost half of all polytraumatized and are expected to grow further in the coming decades [6]. Young and old polytraumatized patients have proved to be different groups in terms of injury pattern, survival and clinical outcome [7, 8]. The young sustain more high-impact injuries yet show a higher survival rate and fewer in-hospital complications than the old.

To improve current polytrauma care, it is important to look beyond mere survival and to assess patient-reported aspects of long-term mental and functional outcome. The literature on the effect of age on patient-reported HRQoL after sustaining a trauma/polytrauma ranges from reporting no effect to a significant negative relationship [3, 5, 9]. However, these studies provided no information on pre-injury status, making the relationship between age and reported outcome hard to interpret.

A polytrauma often leads to permanent life changes and recovery, and rarely reaches the pre-injury state. The ultimate goal for most polytrauma survivors is to fully adapt and embrace life after the injury, including its limitations. This is in line with the new proposed definition of health by Huber et al. from 2011, where health is regarded as the ability to adapt and self-manage [9]. The quality to withstand adversity and bounce back from difficult life events is called resilience [11]. This is a multidimensional personal trait that originates from the field of psychology and is gaining attention in the field of medicine (e.g., oncology and orthopedics). It is relevant because it is measurable, trainable, and for most, it could make the difference in the recovery process after sustaining a major trauma. Multiple studies show that resilience does not decline with age and that the elderly show the same or higher scores on resilience compared to young adults [12, 13].

One way to measure resilience within a trauma population is to assess whether HRQoL has recovered after the major trauma. The research field of acute medicine, however, is limited by the lack of prospective pre-injury screening, complicating the definition of the true impact of a trauma. Normative data of HRQoL surveys could be used as a substitute and may serve as a criterion for resilience [14]. Hence, the main objective of this multicenter study was to explore the effect of age of surviving polytrauma patients on patient-reported HRQoL and their resilience capacity, from 1 up to 5 years post-injury using age- and sex-adjusted normative data.

## Methods

### Study population and design

A multicenter cross-sectional survey study was conducted in 2018, including all surviving adult patients with an ISS ≥ 16 who presented between January 2013 and January 2017 at one of the three participating trauma centers in the northern Netherlands. Patients were identified through the Dutch Trauma Registry (DTR), a mandatory ongoing database based on the Major Trauma Outcome Study (MTOS +) [15]. Patients who had a traceable Dutch home address, who were able to complete a set of Dutch questionnaires and who provided a written informed consent were requested to fill in a set of questionnaires with a follow-up ranging from 1 up to 5 years. Isolated thermal injuries and submersions were excluded. After six weeks, a single reminder was sent to the non-respondents.

For purposes of this study, demographics, injury characteristics (Abbreviated Injury Scale, AIS scores) and physical (Glasgow Coma Scale and systolic blood pressure on admission) and clinical characteristics (admission duration, intensive care admission and duration) were extracted from the DTR database in order to identify possible determinants of patient-reported outcomes. The abbreviated injury scores (AIS, version 2008) were used to divide into the following injured regions: head, thorax, abdomen, spine, upper and lower extremity [16].

### Outcome assessment

In this study, the Dutch EuroQol (EQ-5D-5L) and the Dutch Short Musculoskeletal Function Assessment (SMFA) were used as generic measures to analyze HRQoL. Both patient-reported outcome measures (PROMs) are shown to be valid and reliable for assessing HRQoL of the trauma population [17–19]. Recent national normative data are available for both the EQ-5D-5L and the SMFA, enabling an age- and sex-corrected comparison with the general Dutch population [20, 21].

The generic EQ-5D-5L classification of health covers the main health domains that are affected by injury [22]. In this classification, health is defined along five dimensions: mobility, self-care, usual activities, pain and discomfort, and anxiety and depression. Each dimension has five levels. A domain-related scoring algorithm based on empirical valuations from the Dutch general population and subsequent statistical modeling is available, by which each health status description can be expressed into a utility score (EQ-5D-US), ranging from 1 for perfect health to 0 for death [20].

The SMFA was designed to assess health status and HRQoL of patients with a broad range of musculoskeletal injuries and disorders. It consists of 46 items that are scored on an ordinal five-point Likert scale, which has shown a superior structural validity with the trauma population in a four-subscale structure [17, 18] compared to the initial two-index structure [23]. The SMFA will be used to evaluate four constructs using the subscales “upper extremity dysfunction” (6 items), “lower extremity dysfunction” (12 items), “problems with daily activities” (20 items) and “mental and emotional problems” (8 items). The sum scores of all four subscales are transformed into a score ranging from 0 to 100, where 100 equals the best possible score.

### Statistical analysis

Baseline characteristics of the respondents were split up per specific age decade: 18–29, 30–39, 40–49, 50–59, 60–69, 70–79 and ≥ 80 years. A non-response analysis was performed on demographics, injury characteristics and clinical characteristics. AIS scores were dichotomized for each aforementioned anatomical region with a threshold of AIS ≥ 3, which was considered a severe injury of this specific region. Glasgow Coma Scale (EMV score ≤ 8) and systolic blood pressure (SBP ≤ 90 mmHg) were dichotomized. Categorical variables were presented using frequencies and percentages, and tested using Pearson’s Chi-square test. Normally distributed continuous variables were presented using means and standard deviations and tested with independent-samples T-test. Non-Gaussian distributed variables were presented as median and interquartile range (IQR), and tested with the Kruskal–Wallis test.

Missing data were present in 2.2% of all survey items (EQ-5D-5L: 5/1800 items, SMFA: 395/16560 items). Fully conditional specification multiple imputation was used to handle these missing data as guided by Van Buuren [24]. Missingness at random was assumed and checked. The number of imputations was 20, with a maximum of 10 iterations. Data were imputed using the five individual items of EQ-5D-5L, the sum score of the four each subscales of the SMFA and age.

First, the effect of age (in years) on the EQ-5D-5L utility score and the four SMFA subscale scores was tested with linear regression, corrected for the individual body regions (AIS < / ≥ 3), EMV ≤ / > 8, ICU admission and months of follow-up (method: Enter). Reporting problems on item level of the EQ-5D-5L are analyzed on differences between the age decades with the Chi-square test.

Subsequently, the utility scores of the EQ-5D-5L and the scores of the four SMFA subscales were compared to the age- and sex-adjusted Dutch normative data [20, 21]. Patients were classified as being resilient when their score was within the 95% confidence interval of the age- and sex-adjusted normative score. Normative data for the SMFA are only present up to age 75. Patients aged 75 years and older in this study were therefore compared to the highest age group of the normative data.

Second, the effect of age on being resilient for the EQ-5D-5L and the four SMFA subscales was assessed by means of multivariate logistic regression analysis (Method: Enter, p-removal: 0.157), corrected for the individual severely injured body regions (AIS < / ≥ 3), EMV ≤ / > 8, ICU admission and months of follow-up. Odds ratios (OR) with corresponding 95% confidence intervals are reported.

Multiple imputation and statistical analyses were performed with IBM SPSS Statistics 23.0 (IBM, Armonk, NY). Significance of statistical differences was attributed to *p* < 0.05.

## Results

### Patients

A total of 632 polytrauma survivors who met the inclusion criteria were identified; 363 of them returned a set of completed questionnaires (response rate: 57%). Median time to follow-up was 35 months (IQR: 20–47), mean age 53 years (SD: 17.8) and median ISS 21 (IQR: 17–26), with a majority of males (71%). Among the respondents, 43 patients (11.8%) were aged 75 years and older.

The non-response analysis (Table 1) showed two significant differences. The median age of the non-responders was lower compared to the responding group (non-respondents 45 years vs. respondents 53 years, *p* < 0.01). Besides, the non-respondents had a shorter median admission duration compared to the respondents (non-respondents 11 days vs. respondents 13 days, *p:* 0.04). Gender ratio, months of follow-up, injury characteristics (ISS and AIS ≥ 3 scores) and clinical parameters showed no significant differences.

**Table 1:** Non-responder analysis

	Non-responders (*n* = 269)	Responders (*n* = 363)	*p-*value
Females (%)	73 (27%)	106 (29%)	0.52
Mean age (SD)	45.2	53.4	** < 0.001**
**Injury characteristics**			
Median ISS (IQR)	22 (17–29)	21 (17–26)	0.51
AIS head ≥ 3	143 (53%)	185 (51%)	0.66
AIS thorax ≥ 3	133 (49%)	154 (43%)	0.10
AIS abdomen ≥ 3	35 (13%)	45 (12.5%)	0.85
AIS spine ≥ 3	49 (18%)	50 (14%)	0.14
AIS upper extremity ≥ 3	14 (5%)	25 (7%)	0.37
AIS lower extremity ≥ 3	48 (18%)	64 (18%)	0.98
**Physical parameters on admission**			
Glasgow Coma Scale ≤ 8*	58 (22%)	91 (26%)	0.29
SBP (< 90 mmHg)	15 (16%)	19 (5%)	0.86
**Clinical parameters**			
Median admission duration (IQR)	11 (6–21)	13 (7–22)	**0.04**
ICU admission (% yes)**	150 (58%)	222 (66%)	0.06
Median no. days ICU (IQR)	2 (0–5)	2 (0–5)	0.18

*IQR* interquartile range, *SD* standard deviation, *AIS* abbreviated injury score, *SBP* systolic blood pressure, *ICU* intensive care unit

Significant results are bolded

^*^20 missing, **33 missing

Table 2 shows the baseline characteristics for all respondents, split up per age group. Some significant differences were noted between the different age groups. Higher age was significantly associated with less severe abdominal trauma and less intensive care admissions among polytraumatized patients.

**Table 2:** Baseline characteristics

	Ages 18–29 (*n* = 57)	Ages 30–39 (*n* = 22)	Ages 40–49 (*n* = 48)	Ages 50–59 (*n* = 80)	Ages 60–69 (*n* = 82)	Ages 70–79 (*n* = 56)	Ages ≥ 80 (*n* = 18)	*p-*value
Females (%)	14 (25%)	7 (32%)	12 (25%)	28 (35%)	19 (23%)	21 (38%)	5 (33%)	0.45
Mean age (SD)	23.2 (3.6)	35.1 (2.7)	44.7 (2.5)	54.3 (2.7)	64.0 (2.9)	74.2 (2.5)	83.6 (3.6)	NA
**Injury characteristics**								
Median ISS (IQR)	25 (19–29)	25 (20–29)	22 (17–28)	22 (17–26)	21 (17–26)	21 (17–25)	21 (17–29)	0.15
AIS head ≥ 3	27 (48%)	10 (46%)	23 (48%)	37 (46%)	42 (51%)	37 (66%)	9 (60%)	0.38
AIS thorax ≥ 3	25 (44%)	9 (41%)	26 (54%)	31 (39%)	35 (43%)	20 (36%)	8 (53%)	0.55
AIS abdomen ≥ 3	15 (26%)	6 (27%)	7 (15%)	9 (11%)	4 (5%)	4 (7%)	0 (0%)	** > 0.001**
AIS spine ≥ 3	7 (12%)	5 (23%)	2 (4%)	10 (13%)	14 (17%)	8 (14%)	4 (27%)	0.22
AIS upper extremity ≥ 3	5 (8%)	2 (9%)	5 (10%)	2 (3%)	7 (9%)	4 (7%)	0 (0%)	0.51
AIS lower extremity ≥ 3	15 (26%)	3 (16%)	11 (23%)	12 (15%)	12 (15%)	9 (16%)	2 (13%)	0.51
**Physical parameters on admission**								
Glasgow Coma Scale ≤ 8*	21 (37%)	7 (32%)	11 (23%)	21 (27%)	20 (26%)	8 (15%)	3 (21%)	0.24
SBP (< 90 mmHg)	3 (5%)	2 (9%)	1 (2%)	5 (6%)	6 (7%)	1 (2%)	1 (7%)	0.69
**Clinical parameters**								
Median admission duration (IQR)	11 (7–21)	15.5 (8–30)	13.5 (8–24)	15 (8–23)	11 (6–16)	12.5 (6–19)	16 (10–23)	0.22
ICU admission (% yes)**	44 (79%)	14 (73%)	32 (71%)	52 (70%)	43 (57%)	28 (52%)	9 (64%)	**0.04**
Median no. days ICU (IQR)	2 (1–4.75)	4 (0–14)	2 (0–6)	3 (0–7)	2 (0–40)	1 (0–3)	4.5 (0–10)	** > 0.001**

*IQR* interquartile range, *SD* standard deviation, *AIS* abbreviated injury score, *SBP* systolic blood pressure, *ICU* intensive care unit

Significant results are bolded

^*^11 missing, **22 missing

### Effect of age on patient-reported outcome

**Fig. 1:**
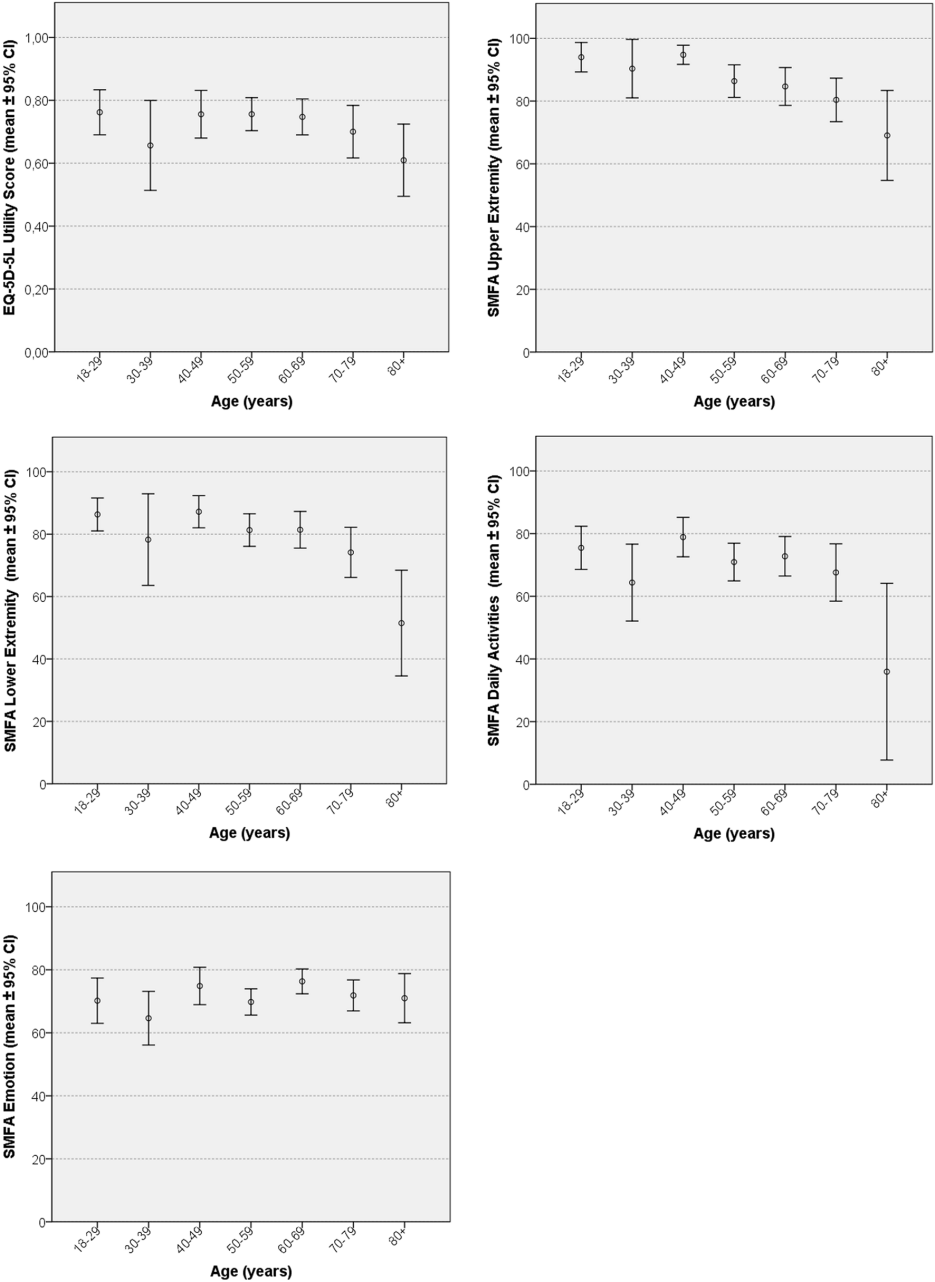
Standardized EQ-5D-5L utility score and SMFA subscales scores for different age decades

Figure 1 displays the mean standardized scores with the corresponding standard deviation interval of the study per age decade. For comparison’s sake, the mean scores of all surveys and reporting of problems at the item level of the EQ-5D-5L are presented for each specific age decade in Table 3.

**Table 3:** Scores of EQ-5D-5L, reporting problems on the EQ-5D-5L items and SMFA subscales

	Ages 18–29 (*n* = 57)	Ages 30–39 (*n* = 22)	Ages 40–49 (*n* = 48)		Ages 50–59 (*n* = 80)	Ages 60–69 (*n* = 82)	Ages 70–79 (*n* = 56)	Ages ≥ 80 (*n* = 18)	*p*-value
**EuroQoL (EQ-5D-5L)**									
Mean EQ-5D-US (SD)	0.76 (0.27)	0.65 (0.32)	0.76 (0.26)		0.75 (0.24)	0.75 (0.31)	0.70 (0.21)	0.61 (0.27)	*
Reporting problems on EQ-5D-5L									
Mobility	20 (35%)	10 (46%)	20 (42%)		31 (39%)	37 (45%)	36 (64%)	12 (80%)	**0.04**
Self-care	10 (18%)	7 (32%)	10 (21%)		21 (26%)	24 (29%)	20 (36%)	13 (69%)	** > 0.001**
Daily activities	29 (51%)	15 (68%)	28 (58%)		48 (60%)	40 (49%)	32 (57%)	14 (93%)	**0.05**
Pain and discomfort	33 (58%)	18 (82%)	33 (69%)		55 (69%)	53 (65%)	36 (64%)	13 (87%)	0.28
Anxiety and depression	21 (37%)	9 (41%)	18 (38%)		31 (39%)	28 (34%)	21 (38%)	7 (47%)	0.92
**Mean standardized scores SMFA subscales**									
Upper extremity dysfunction (SD)	94.0 (17.2)	90.3 (21.0)	94.7 (10.2)		86.4 (22.9)	84.7 (27.0)	80.4 (25.7)	69.1 (24.8)	*
Lower extremity dysfunction (SD)	86.3 (19.3)	78.3 (27.5)	87.2 (16.1)		81.3 (22.7)	81.4 (24.5)	74.2 (25.8)	51.5 (18.3)	*
Daily activities (SD)	75.5 (25.1)	64.4 (27.7)	78.9 (20.4)		70.9 (25.6)	72.8 (26.9)	67.6 (27.8)	35.9 (17.7)	*
Emotion (SD)	70.2 (25.8)	64.6 (19.2)	74.8 (19.7)		69.8 (18.5)	76.3 (17.7)	71.9 (17.5)	71.0 (13.5)	*

^*^See text for linear regression analysis

#### EQ-5D-5L

Overall, the polytraumatized patients had a mean EQ-5D-5L utility score of 0.73, with scores ranging from 0.61 (age group ≥ 80) to 0.76 (age groups 18–29 and 40–49). In total, 46.2% of all patients reported problems with mobility, 29.2% with self-care, 57.2% with daily activities, 67.5% reported a form of pain or discomfort, and 37.2% reported mental problems with anxiety or depression. No significant effect of age was found on the utility score. Higher age did not affect the utility score significantly (B: – 0.001, *p*: 0.15, CI: – 0.03–0.00).

As shown in Table 3, higher age was significantly associated with reporting problems on the EQ-5D-5L items: mobility, self-care and daily activities. No significant association was found for the items pain and discomfort or anxiety and depression.

#### SMFA

For SFMA scores, a significant effect for higher age was seen within upper extremity dysfunction (B: – 0.29, *p* < 0.001, CI: – 0.42 to –0.16), lower extremity dysfunction (B: -0.30, *p* < 0.001, CI: – 0.45 to – 0.16) and daily activities subscales (B: – 0.21, *p*: 0.01, CI: – 0.37 to – 0.05). No significant trend for age was found on the standardized scores for mental and emotional problems (B: 0.047, *p*: 0.47, CI: – 0.08–0.18).

### Effect of age on resilience

**Fig. 2:**
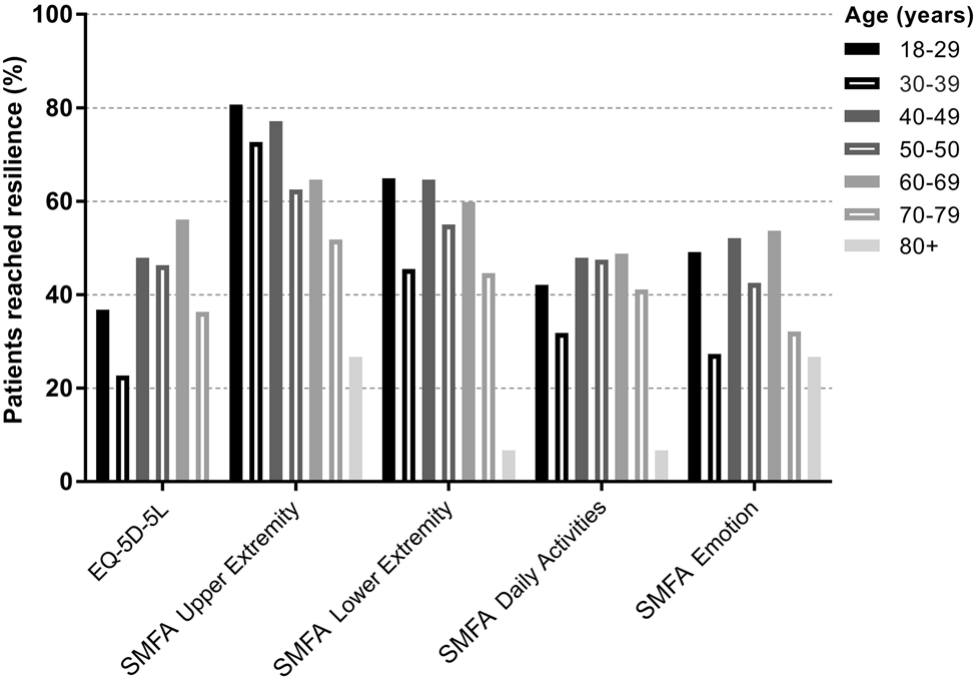
Resilience on subscales SMFA and EQ-5D-5L utility score for the different age decades

Figure 2 and Table 4 present the percentages of the study population classified as being resilient (having reached the 95% confidence interval of their age- and sex-matched peers of the general Dutch population) on the four subscales of the SMFA and the utility score of the EQ-5D-5L, per age group.

**Table 4:** Resilience on subscales SMFA and EQ-5D-5L utility score for the different age decades

Age (years)	*n*	EQ-5D-5L	SMFA			
			Upper extremity	Lower extremity	Daily activities	Emotion
18–29	57	37%	81%	65%	42%	49%
30–39	22	23%	73%	46%	32%	27%
40–49	48	48%	77%	65%	48%	52%
50–59	80	46%	63%	55%	48%	43%
60–69	82	56%	65%	60%	49%	54%
70–79	56	36%	52%	45%	41%	32%
≥ 80	18	0%	28%	7%	7%	27%
Total	363	42%	65%	54%	44%	44%

^*^Imputed data are presented

#### EQ-5D-5L

Less than half (42%) of the polytrauma population was classified as being resilient in their HRQoL, based on the EQ-5D-5L utility score. Polytrauma patients in their sixties showed the highest resilience (56%), closely followed by those in their forties (47%) and fifties (46%). No octogenarians showed resilience in the EQ-5D-5L. A decline in reaching the Dutch norm of HRQoL as measured with the EQ-5D-5L was seen not only for the very old, but also for the youngest age cohorts, especially polytrauma patients aged 30–39, only 22% of whom reached resilience. Patients aged 60–69 showed significantly higher resilience compared to the reference group (OR: 2.20, CI: 1.49–3.27, *p:* 0.04, Table 5). Other age groups did not show differences in resilience compared to the reference group.

**Table 5:** Effect of age on reaching resilience using binary logistic regression

Age (years)	*n*	Odds ratio	95% CI		*p*-value
			Lower	Upper	
**EQ-5D-5L**					
18–29*	57	–	–	–	–
30–39	22	0.51	0.14	1.79	0.30
40–49	48	1.28	0.56	2.90	0.57
50–59	80	1.50	1.02	2.20	0.41
60–69	82	2.20	1.49	3.27	**0.04**
70–79	56	0.68	0.29	1.60	0.38
≥ 80	18	0.00	0.00	0.00	1.00
**Upper extremity (SMFA)**					
18–29*	57	–	–	–	**–**
30–39	22	0.75	0.21	2.62	0.65
40–49	48	0.50	0.18	1.41	0.19
50–59	80	0.33	0.13	0.79	**0.01**
60–69	82	0.35	0.14	0.87	**0.02**
70–79	56	0.15	0.06	0.40	** > 0.01**
≥ 80	18	0.06	0.01	0.29	** > 0.01**
**Lower extremity (SMFA)**					
18–29*	57	–	–	–	–
30–39	22	0.39	0.12	1.24	0.11
40–49	48	0.65	0.27	1.57	0.34
50–59	80	0.57	0.26	1.24	0.15
60–69	82	0.71	0.32	1.58	0.40
70–79	56	0.26	0.11	0.63	** > 0.001**
≥ 80	18	0.07	0.01	0.55	** > 0.001**
**Daily activities (SMFA)**					
18–29*	57	–	–	–	–
30–39	22	0.48	0.15	1.59	0.23
40–49	48	1.02	0.44	2.37	0.97
50–59	80	1.14	0.53	2.44	0.73
60–69	82	1.29	0.60	2.78	0.52
70–79	56	0.56	0.24	1.33	0.19
≥ 80	18	0.18	0.02	1.53	0.12
**Emotion (SMFA)**					
18–29*	57	–	–	–	–
30–39	22	0.28	0.08	0.99	**0.04**
40–49	48	0.77	0.33	1.76	0.53
50–59	80	0.67	0.32	1.41	0.29
60–69	82	1.03	0.49	2.18	0.94
70–79	56	0.37	0.16	0.86	**0.02**
≥ 80	18	0.44	0.11	1.76	0.25

Imputed data are presented

Significant results are bolded

^*^Reference age category

All survey scores are corrected for the individual severely injured body regions (AIS ≥ 3), EMV ≤ 8, ICU admission and months of follow-up

#### SMFA

Considering resilience on the upper extremity and lower extremity dysfunction subscales, a clear declining trend was seen for higher age (Fig. 2). This is confirmed by the results of the binary logistic regression analysis (as displayed in Table 5), where higher age from ≥ 50 years (OR: 0.33, CI: 0.13–0.79, *p*: 0.01) for the upper extremity and ≥ 70 years for the lower extremity (OR: 0.26, CI: 0.11–0.63, *p* < 0.001) is significantly associated with a reduced resilience on these subscales.

In total, 46.3% of the total study population showed resilience on the daily activities subscale. No significant differences were found between the age groups. Patients aged 60–69 showed the highest resilience (53.7%) on the mental and emotional problems subscale. Octogenarians (26.7%), tricenarians (27.3%) and septuagenarians (32%) showed the lowest resilience. Tricenarians and septuagenarians showed significant lower odds for resilience on this subscale compared to the youngest group (30–39 years: OR: 0.28, CI: 0.08–0.99, *p*: 0.04 and 70–79 years: OR: 0.37, CI: 0.16–0.86, *p*: 0.02, respectively).

## Discussion

The main goal of this study was to examine the effect of age on health-related quality of life and the capacity to show resilience, in this study defined as reaching the age- and sex-adjusted normative HRQoL. The explanation of the role of age in this matter requires a multilayered answer.

Polytrauma survivors in our population overall have undeniably lower HRQoL (as measured with the EQ-5D-5L, mean: 0.73) compared to the healthy population (mean: 0.87–0.88) [20, 25]. Our findings are in line with the results of a recent Dutch study, having a comparable mean utility score at one year of follow-up after a severe trauma [26]. To put the burden of a polytrauma survivor in perspective, the presented range of HRQoL in this study measured with the EQ-5D-5L is comparable to patients with severe COPD, cardiovascular disease and multiple sclerosis [20, 27, 28].

Considering the effect of age, there is a clear difference in performance on the physical and non-physical aspects of HRQoL. Elderly report significantly worse functioning of the upper and lower extremities after sustaining a polytrauma compared to younger persons. By contrast, emotional well-being, pain and discomfort, and anxiety and depression were not clearly affected by increasing age. Elderly likewise report more problems with mobility, self-care and daily activities. But to what extent does the found difference in this study relate to the trauma sustained from the injury, instead of just aging? Mobility, self-care ability and performance of the legs and arms will inevitably decline by a certain age. A comparable yet less distinct pattern for increasing age is seen in the normative data of the Dutch population of the SMFA [21]. To fully understand the impact of age on the quality of life of a surviving polytrauma patient, this must be put in the perspective of the normative data.

Overall, less than half of the study population showed resilience on HRQoL measured with the EQ-5D-5L. A cautious statement would be that probably the majority of polytrauma survivors had to give in on their quality of life. The young, however, were able to reach higher standards of resilience, mainly on the physical aspects of HRQoL. Only resilience in physical functioning of the upper and lower extremities was affected by age, with higher age associated with lower odds of being resilient. There was a significant association with increasing age on performing less well on daily activities, yet no significant differences were found for resilience between the age categories on this particular aspect—hence, surviving a polytrauma does not impact the elderly more than the young. Moreover, polytrauma survivors in their sixties showed doubled resilience on the HRQoL (as measured with the EQ-5D-5L) compared to the group of younger adults (aged 18–29). This suggests that the elderly could show the same, if not higher levels of resilience on some of the non-physical dimensions of HRQoL compared to the younger cohorts. An exception must be made for octogenarians, although this was a small group, who showed the lowest levels of resilience on all outcomes of all age groups. These findings are in line with the results presented by Gross et al. in 2018 [29]. They concluded that the differences between young and old polytrauma patients are mainly explained by the low-achieving octogenarians. Supporting this difference in outcome is a study conducted by Hopman in 2009. Among the chronically ill an equivalent pattern is seen where elderly scored lower on the physical components of HRQoL and better on the mental components, compared to the younger age groups [30].

A remarkable finding in this study are the results for patients in their thirties. They seem to report lower scores on the non-physical dimensions of HRQoL compared to their neighboring age categories and lower rates of resilience for all reported outcomes.

According to the data provided by this study, no clear explanation for this finding can be stated, as no differences were found in demographics or injury characteristics for this particular age group. It may be explained using a more holistic perspective. Tricenarians are on the footstep of their lives. This category of young adults is characterized by several big transitions: planning a family, making a career through job advancement and choosing a place to settle. A recent study showed only 68% of all polytrauma survivors returned to work after one year, 31% partially [26]. Work resumption was also found to be an important factor related to experienced quality of life among the severely injured [31]. Tricenarians’ expectations and demands are more explicit than those of younger adults and, compared to the old, often not yet fulfilled. Sustaining a major trauma in this tumultuous period of life may therefore be more difficult to overcome. This is also suggested by the results of Terril et al., where middle-aged and younger participants with disabling medical conditions showed the lowest levels of resilience [32]. That age cohort should therefore be of special interest to future research, as they are particularly young and could benefit from potentially large gains.

Besides age, injury pattern could be an important factor on resilience. Sustaining a traumatic brain injury or spinal cord injury often leads to a serious decline in HRQoL [33, 34]. In 2016, a multicenter study examined resilience at 3 months post-injury, relying on a sample of adults with moderate to severe TBI, and provided evidence that resilience levels were relatively low in comparison with the general population [35]. Moreover, personality, social support, reported pain and anxiety are also known factors which influence the ability to bounce back and show resilience [36, 37]. These findings represent an opportunity for future research and targeted intervention to increase resilience of at-risk groups.

The study findings have important implications for clinicians and researchers, most notably that resilience should be considered as an important factor in the aspects of outcome and revalidation after sustaining a major trauma. To our knowledge, this is the first multicenter study to obtain HRQoL of polytrauma survivors linked to age- and sex-matched normative data in a first attempt to measure resilience. This study pointed out that normative data, and moreover resilience, could be used to obtain a more palpable effect on patient-reported outcome where no pre-injury data are available. Normative data could prove more useful in future research as its availability on many surveys grows. Resilience is affected by age, but also strongly depends on the different aspects of HRQoL (physical vs. non-physical). Research that considers analyzing other resilience-related factors helps early identification of patients who could benefit from intensified rehabilitation.

Some limitations of this study should be mentioned. Caution is advised, as normative data are not a fully reliable substitute for pre-injury functioning. Trauma populations have higher preexisting comorbidities than non-injured populations [38]. This could lead to an overestimation of the reported problems and, for this study, an underestimation of the presented resilience. Still, a recent study pointed out that there was no clinically relevant difference in patient-reported HRQoL between the Dutch population and a prospectively collected pre-injury trauma population [14]. Another limitation of this study is the response rate of 57%, although it is in line with other studies on trauma populations. The response rate among the young in our study was lower compared to the old, which could lead to a underrepresentation of the young within the study. The injury characteristics and clinical parameters between the respondent groups, however, did not differ, even when split up per age group. Therefore, the cause of the higher non-response of the young remains unclear.

## Conclusion

The majority of polytrauma survivors do not recover fully. In the light of outcome after surviving a major trauma, binary comparison between young and old is not always a valid one. A clear negative effect with higher age is seen for reported physical outcome. No linear or binary relationship with age was seen for the non-physical aspects of HRQoL. A decline on almost all aspects of HRQoL was seen from age 70, whereas very few octogenarians have the capacity to recover from a polytrauma. This makes them the most vulnerable group. And yet in contrast to the very old and to a lesser extent tricenarians, sexagenarians are overall one of the most resilient groups after surviving a polytrauma.
